# Campylobacter devanensis sp. nov., Campylobacter porcelli sp. nov., and Campylobacter vicugnae sp. nov., three novel Campylobacter lanienae-like species recovered from swine, small ruminants, and camelids

**DOI:** 10.1099/ijsem.0.006405

**Published:** 2024-06-06

**Authors:** William G. Miller, Bruno S. Lopes, Meenakshi Ramjee, Michele T. Jay-Russell, Mary H. Chapman, Tina G. Williams, Delilah F. Wood, Igor Gruntar, Bojan Papić, Ken J. Forbes

**Affiliations:** 1 Produce Safety and Microbiology Research Unit, Agricultural Research Service, U.S. Department of Agriculture, Albany, CA, USA; 2 School of Health and Life Sciences, Teesside University, Middlesbrough, UK; 3 National Horizons Centre, Teesside University, Darlington, UK; 4 Wolfson Wohl Cancer Research Centre, Glasgow. The University of Glasgow, Glasgow, UK; 5 Western Center for Food Safety, University of California, Davis, CA, USA; 6 Bioproducts Research Unit, Agricultural Research Service, U.S. Department of Agriculture, Albany, CA, USA; 7 University of Ljubljana, Veterinary Faculty, Institute of Microbiology and Parasitology, Ljubljana, Slovenia; 8 School of Medicine, Medical Sciences and Nutrition, University of Aberdeen, Aberdeen, UK

**Keywords:** alpaca, *Campylobacter*, goat, novel species, sheep, swine

## Abstract

In a previous study characterizing *Campylobacter* strains deficient in selenium metabolism, 50 strains were found to be similar to, but distinct from, the selenonegative species *Campylobacter lanienae*. Initial characterization based on multilocus sequence typing and the phylogeny of a set of 20 core genes determined that these strains form three putative taxa within the selenonegative cluster. A polyphasic study was undertaken here to further clarify their taxonomic position within the genus. The 50 selenonegative strains underwent phylogenetic analyses based on the sequences of the 16S rRNA gene and an expanded set of 330 core genes. Standard phenotypic testing was also performed. All strains were microaerobic and anaerobic, Gram-negative, spiral or curved cells with some displaying coccoid morphologies. Strains were motile, oxidase, catalase, and alkaline phosphatase positive, urease negative, and reduced nitrate. Strains within each clade had unique phenotypic profiles that distinguished them from other members of the genus. Core genome phylogeny clearly placed the 50 strains into three clades. Pairwise average nucleotide identity and digital DNA–DNA hybridization values were all below the recommended cut-offs for species delineation with respect to *C. lanienae* and other related *Campylobacter* species. The data presented here clearly show that these strains represent three novel species within the genus, for which the names *Campylobacter devanensis* sp. nov. (type strain RM3662^T^=LMG 33097^T^=NCTC 15074^T^), *Campylobacter porcelli* sp. nov. (type strain RM6137^T^=LMG 33098^T^=CCUG 77054^T^=NCTC 15075^T^) and *Campylobacter vicugnae* sp. nov. (type strain RM12175^T^=LMG 33099^T^=CCUG 77055^T^=NCTC 15076^T^) are proposed.

## Data Summary

Four supplementary figures, five supplementary tables and one supplementary file are provided in the online version of this article. All supplementary data is available through Figshare at https://doi.org/10.6084/m9.figshare.25796689.

## Introduction

*Campylobacter* species are the leading cause of bacterial gastroenteritis in humans worldwide [1]. At the time of writing, the genus *Campylobacter* currently comprises 45 validly described species [2]. These species can be phenotypically divided into two groups: the ‘thermotolerant’ *Campylobacter*, which can grow up to 42 °C, and the ‘non-thermotolerant’ *Campylobacter*, in which the optimal growth temperature generally does not exceed 37 °C. This phenotypic grouping is reflected in the whole-genome phylogeny, which clearly splits *Campylobacter* taxa into two major branches [3]. The signature taxa in the thermotolerant branch are *Campylobacter jejuni* and *Campylobacter coli*, which are responsible for most of the reported *Campylobacter*-associated human illnesses [4]. Thermotolerant campylobacters have a wide host range and are recovered from wild and domesticated warm-blooded animals, especially poultry [5,6]. In contrast, non-thermotolerant campylobacters have a narrower host range and are recovered mainly from humans, livestock, and occasionally from other animal species such as reptiles [5]. One particular branch within the non-thermotolerant division is the *Campylobacter fetus* group, which contains five species, namely *C. fetus* [7], *C. hyointestinalis* [8], *C. lanienae* [9], *C. iguaniorum* [10] and *C. magnus* [11]. *C. fetus* and *C. hyointestinalis* are further subdivided into three and two subspecies, respectively [12,14]. The typical host range for this group includes warm-blooded animals such as swine, cattle, deer, and sheep [5,15,17]. Three *C. fetus* group taxa, namely *C. fetus* subsp. *testudinum*, *C. hyointestinalis* and *C. iguaniorum*, have been recovered from reptiles [10,12, 18]; *C. iguaniorum* was also isolated from an alpaca [19]. Some taxa within this group have occasionally been associated with human illness [5,20, 21].

In 2017, Miller *et al*. [22] identified a discrete cluster of related strains within the *C. fetus* group that is deficient in selenium metabolism. Genome-wide comparison of these strains showed that the members of this cluster lack the genes encoding the selenocysteine insertion machinery, selenoproteins (e.g., formate dehydrogenase) and the selenocysteinyl tRNA. In addition, these strains also possess four to ten non-identical and genetically unlinked flagellin genes. Although *C. lanienae* was the primary species within this selenonegative group, strain typing using *atpA* [23] suggested that 50 selenonegative strains were related to, but distinct from, *C. lanienae*. These strains were further characterized by multilocus sequence typing (MLST) and core genome analysis [22]. MLST analysis, using the *C. lanienae* PubMLST scheme [24], identified 48 different sequence types (Table S1), suggesting substantial genetic diversity. A dendrogram based on the concatenated MLST profiles showed that the 50 strains form three distinct clades within the selenonegative group. This phylogenetic structure was also supported by a dendrogram based on the alignment of 20 concatenated core protein sequences. The first novel taxon was named clade 1 [22] and is represented by strain NCTC 13003 (redeposited into NCTC in this study as NCTC 15074^T^). The isolation source was listed as ‘Unknown’ in [22]; however, NCTC 15074^T^ was subsequently determined to have been isolated in 1994 from pig faeces in Switzerland (Table S1). The second novel taxon was named clade 2 [22] and is represented by strain RM6137^T^, which was isolated from wild boar faeces in 2006 in California, USA (Table S1). The third novel taxon was named clade 3 [22] and is represented by strains RM12175^T^ and RM8964. Both strains were isolated in California, USA, in 2010 and 2009, respectively (Table S1). RM8964 was recovered from goat faeces. The isolation source for RM12175^T^ was listed in Miller *et al*. [22] as cow faeces; however, it was later determined that this strain was actually recovered from alpaca faeces. Subsequent to Miller *et al*. [22], five additional strains were recovered following sampling of dead pigs at slaughterhouses in Slovenia: two belonged to clade 1, whereas the other three belonged to clade 2. A recent study by Wang *et al*. [25] described three strains belonging to clade 3, along with the strains described here and also previously in Miller *et al*. [22]. They proposed that these strains belong to a novel *Campylobacter* species, for which they proposed the name ‘*Campylobacter ovis* sp. nov.’. This species is currently not listed as validly published in the List of Prokaryotic names with Standing in Nomenclature [2]. In addition, the proposed type strain for this taxon (GDMCC 1.3685) has only been deposited into a single culture collection and is not accessible from there. Therefore, although ‘*C. ovis* sp. nov.’ has been effectively published, it is not validly published.

In this study, we present a polyphasic analysis of these three clades, which clearly shows that they represent novel species within *Campylobacter*. Since most of the clade 1 strains were first characterized at the University of Aberdeen, the name *Campylobacter devanensis* sp. nov. is proposed, which refers to the Roman name for Aberdeen, i.e., Devana. The name *Campylobacter porcelli* sp. nov. is proposed for clade 2 strains, which were exclusively isolated from swine. The name *Campylobacter vicugnae* sp. nov. is proposed for clade 3 strains since the prospective type strain (RM12175^T^) was recovered from an alpaca (*Vicugna pacos*).

## Morphology

**Fig. 1.**
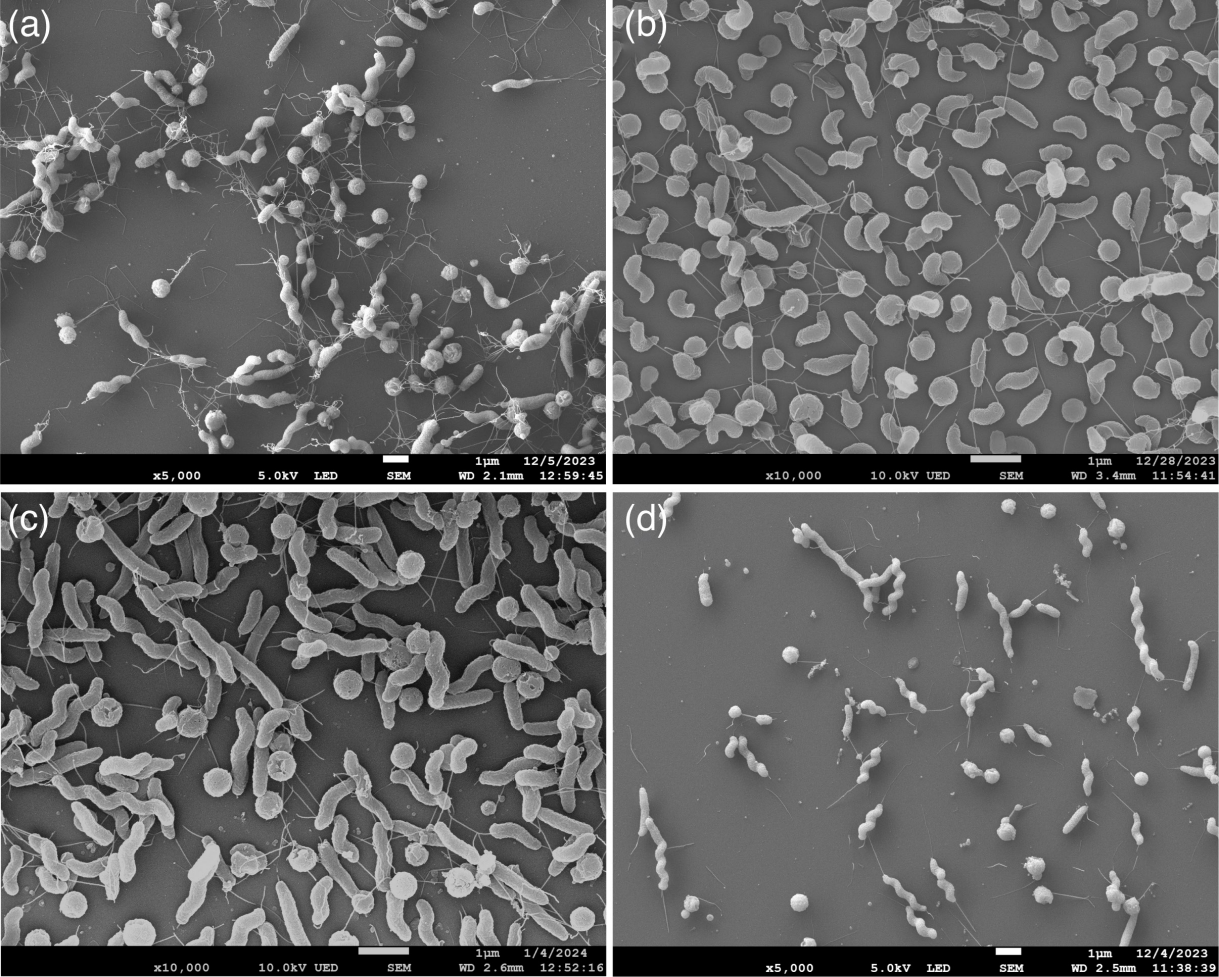
Scanning electron microscope images of (**a**) *Campylobacter devanensis* sp. nov. LMG 33097^T^, (**b**) *Campylobacter porcelli* sp. nov. LMG 33098^T^, (**c**) *Campylobacter vicugnae* sp. nov. LMG 33099^T^ and (**d**) *Campylobacter vicugnae* sp. nov. RM8964 at ×5000 (**a, d**) or ×10000 (**b, c**) magnification.

Cell morphology of strains representing the three novel taxa was determined by scanning electron microscopy (Fig. 1). Bacterial cultures were fixed overnight in 3.84 % (w/v) paraformaldehyde, 2 % (w/v) glutaraldehyde in 0.096 M sodium phosphate buffer, pH 7.2. Cells were loaded onto glass coverslips, pretreated with 0.1 % (w/v) poly-l-lysine for 30 min and washed with phosphate buffer. Coverslips were dehydrated in a graded series of ethanol (50, 70, 80, 95, and 100 %). The coverslips were critical point dried in a Tousimis Autosamdri-815 critical point dryer. Coverslips were mounted onto aluminium specimen stubs using double adhesive coated carbon tabs (Ted Pella) and then coated with platinum in a Leica EM ACE600 (Leica Microsystems) sputter-coating unit. The samples were examined using a jeol JSM-7900F field emission scanning electron microscope. *C. devanensis* and *C. vicugnae* cells were spiral shaped with flagella on both ends, although the helical pitch of the cells varied between strains and cells. *C. porcelli* cells were primarily curved rods with flagella detected only on one end, although some spiral cells with bipolar flagella were also present. Coccoid cells were observed in all cultures. Notably, putative bacteriophage particles were detected in *C. vicugnae* strain RM8964 cultures (Fig. S1). The genome of this strain contains an integrated prophage element, and additional work is ongoing to determine the nature of the putative bacteriophage particles detected here.

## Phenotypic characterization

Strains representing the three putative novel taxa were characterized using standard phenotypic tests [26]. Methods and the control strains used for each test are provided in File S1. Three, six and nine strains of *C. devanensis, C. porcelli* and *C. vicugnae*, respectively, were tested. Strains were tested for: growth at 30 °C, 37 °C and 42 °C under aerobic, microaerobic and anaerobic conditions; motility; oxidase, catalase, nitrate reductase, urease, alkaline phosphatase and hippuricase activity; the ability to hydrolyse indoxyl acetate, and reduce selenite and triphenyltetrazolium chloride (TTC); growth on modified charcoal–cefoperazone–deoxycholate agar (mCCDA) plates or media supplemented with 2 % (w/v) NaCl, 1 % (w/v) glycine or 0.04 % (w/v) TTC; H_2_S production on triple sugar iron (TSI) agar; α-haemolysis on anaerobe basal agar (ABA; Oxoid) amended with 5 % lysed horse blood (Innovative Research), termed hereafter as ABA-B; and resistance to 30 mg l^−1^ cephalothin or 30 mg l^−1^ nalidixic acid. All tests were performed in triplicate, using appropriate positive and negative controls. Additional phenotypic tests for related species that were listed as ‘unknown’ or ‘not tested’ in previous studies were performed here. The results of these tests are presented in Table 1. All three putative novel taxa: could grow under microaerobic or anaerobic conditions at 37 °C and 42 °C, but not aerobically; were motile; were oxidase, catalase, and alkaline phosphatase positive, and urease and hippuricase negative; and could reduce both nitrate and TTC. The absence of α-haemolysis on ABA-B distinguishes all three novel taxa from their closest relative *C. lanienae*. Furthermore, indoxyl acetate hydrolysis and selenite reduction can be used to separate *C. devanensis* (IA^+^/Sel^+^), *C. porcelli* (IA^–^/Sel^+^) and *C. vicugnae* (IA^–^/Sel^–^) strains. Taken together, the three novel selenonegative taxa have phenotypic profiles that are distinct from each other and the other validly described *Campylobacter* species (Tables 1 and S2).

**Table 1.** Phenotypic characteristics of *Campylobacter devanensis* sp. nov., *Campylobacter porcelli* sp. nov., *Campylobacter vicugnae* sp. nov., and related *Campylobacter* species Species: 1, *Campylobacter devanensis* sp. nov. (*n*=3); 2, *Campylobacter porcelli* sp. nov. (*n*=6); 3, *Campylobacter vicugnae* sp. nov. (*n*=9); 4, *Campylobacter lanienae*; 5, *Campylobacter fetus* subsp. *fetus*; 6, *Campylobacter fetus* subsp. *venerealis*; 7, *Campylobacter fetus* subsp. *testudinum*; 8, *Campylobacter hyointestinalis* subsp. *hyointestinalis*; 9, *Campylobacter hyointestinalis* subsp. *lawsonii*; 10, *Campylobacter iguaniorum*; 11, *Campylobacter magnus*; Positive strains: + (95–100 %); M (70–95 %); V (30–70 %); F (10–30 %); − (0–10 %); w, weak growth; for antibiotic resistance, S/V/R indicates sensitive, variable or resistant, respectively. The complete comparison within the genus *Campylobacter* is shown in Table S2. Data for columns 4–11 are derived from the original species descriptions and/or On *et al.* [26], and updated where applicable (^†^).

Characteristic	1	2	3	4	5	6	7	8	9	10	11
Motility	+	+	+	+	+	+	+	+	+	+	+
Oxidase	+	+	+	+	+	+	+	+	+	+	+
Catalase	+	+	+	+	+	+	+	+	+	+	+
Nitrate reduction	+	+	+	+	+	M	+	+	+	+	+
Indoxyl acetate hydrolysis	+	−	−	−	−	−	−	−	−	−	+
Urease activity	−	−	−	−	−	−	−	−	−	−	−
Alkaline phosphatase	+	+	+	+	−	−	−	−	F	+^†^	V
Hippuricase activity	−	−	−	−	−	−	−	−	−	−	−
Selenite reduction	+	+	−	V^†^	M	F	+^†^	+	+	−^†^	+^†^
H_2_S production on TSI	−	−	−	−	−	−	−	+	+	+	−
α-Haemolysis	−	−	−	+	−	V	−^†^	V	V	+	−^†^
S-layer	−	−	−	−	+	+	+	−	−	−	−
Growth in/at/on:											
30 °C (microaerobic)	−	−	−	−	+	+^†^	+	+	+	+	−^†^
37 °C (microaerobic)	+	+	+	+	+	+	+	+	+	+	+
42 °C (microaerobic)	+	+	+	+	M	−	M	+	+	−	+
37 °C (aerobic)	−	−	−	−	−	−	F	−	−	−	−
37 °C (anaerobic)	+	+	+	w	V	M	+	−	+	w	+^†^
2 % NaCl	+	+	−	−^†^	−	−	−^†^	−	−	−^†^	−
1 % Glycine	+	+	−	−	+	F	+	+	F	+	−
0.04 % TTC	−	−	−	V^†^	−	−	+	F	−	−^†^	+^†^
mCCDA	+	+	+	+^†^	+	+	+^†^	+	+	+	+
TTC reduction	+	+	+	+^†^	−	−	+	F	−	+^†^	−
Requirement for H_2_	−	−	−	−	−	−	−	−	−	−	−
Resistance to:											
Nalidixic acid (30 mg l^–1^)	R	R	R	R	R	V	R^†^	R	R	R	R
Cephalothin (30 mg l^–1^)	R	R	V	R	S	S	R^†^	V	S	S	R

## Genome features and 16S rRNA gene intervening sequences

Gap-free, complete genomes of the three proposed type strains were obtained using a hybrid sequencing approach as described previously [22]. In addition, the genomes of the remaining strains were all sequenced to completion (*C. vicugnae* strains RM8835, RM8964 and RM9261) or to draft level using either a hybrid PacBio/Illumina assembly or an Illumina assembly only, respectively ([22] and data not shown). Genome sequencing metrics and accession numbers are provided in Miller *et al*. [22] and in Table S1. The sizes of the completed genomes ranged between 1.58 and 1.73 Mb and are thus on the lower end of the scale for non-thermotolerant *Campylobacter* genomes, but similar in size to the genome of the *C. lanienae* type strain (1.59 Mb). Each putative novel taxon has a distinct DNA G+C content relative to the related *C. lanienae* (*n*=27; 34.46±0.04 mol%): the G+C content for *C. devanensis* (*n*=37) was 33.50±0.02 mol%, whereas the G+C contents for *C. porcelli* (*n*=7) and *C. vicugnae* (*n*=10) were 34.15±0.02 and 32.32±0.04 mol%, respectively. Small (4–6 kb) to medium (25–26 kb) sized plasmids were identified in the complete genomes of *C. porcelli* and *C. vicugnae*. These plasmids were identified during the assembly process as additional non-chromosomal contigs that circularized and contained genes typically associated with small plasmids, e.g., *rep* and *mob*. No noteworthy genes other than those encoding proteins putatively involved in replication or conjugative transfer were identified on these plasmids.

A notable feature of the *C. porcelli* strain LMG 33098^T^ genome is the presence of heterogeneous rRNA loci; the 16S rRNA gene in two loci is 1742 bp with the third 16S rRNA gene having a more typical size of 1512 bp. The larger 16S rRNA gene size is due to a structured intervening sequence (IVS) in helix 10 (h10) of the 5′ major domain (Fig. S2). Structured IVSs of similar size at the same position within h10 have been observed in other *Campylobacter* species such as *C. magnus*, *C. sputorum*, *C. rectus*, * C. helveticus* and *C. curvus* (Fig. S2 and [27,29]). Although the genome of strain LMG 33098^T^ was fully completed, the remaining *C. porcelli* genomes were only sequenced to draft level. To characterize the 16S rRNA loci of these strains, the conserved 16S rRNA gene sequences downstream of h10 were extracted and extended using the Illumina reads. Most of the *C. porcelli* strains examined have homogeneous rRNA loci, in which all three 16S rRNA genes contain IVSs. *C. porcelli* strain CX2-4080-23 contains only 1512 bp 16S rRNA genes, and strain P0078 is completely heterogeneous at the 16S rRNA locus, in which the two larger 16S rRNA genes contain different IVS sequences (Fig. S2). The *Campylobacter* IVSs are generally taxon-specific, with each species having distinct IVSs (Fig. S2 and [27]). Therefore, it is interesting to note that the IVS of P0078 16S rRNA1 is nearly identical to the IVS of *C. magnus* (Fig. S2), suggesting that lateral transfer occurred between the two species. The *C. porcelli* IVSs contain no obvious open reading frames; thus, the role of these IVSs in *C. porcelli* biology remains undetermined.

## Phylogenetic analysis

**Fig. 2.**
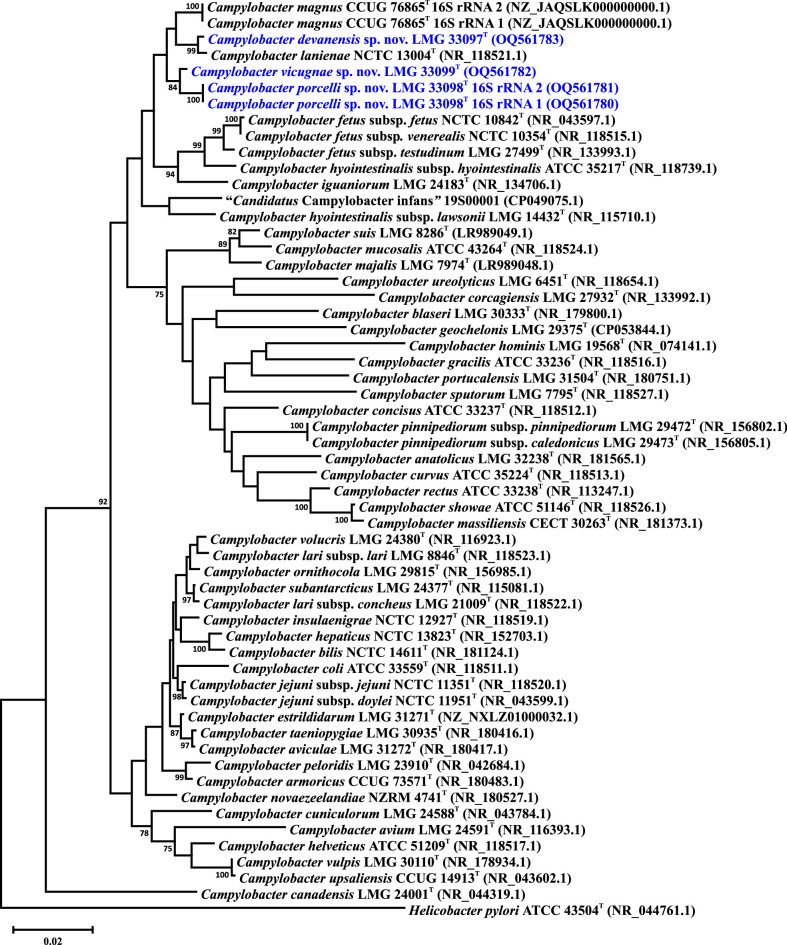
16S rRNA gene phylogenetic tree showing the positioning of *Campylobacter devanensis* sp. nov., *Campylobacter porcelli* sp. nov., and *Campylobacter vicugnae* sp. nov. within the genus *Campylobacter*. Evolutionary history was inferred using the neighbour-joining method [32] and distances were computed using the Kimura two-parameter model [33]. Bootstrap values of ≥75%, generated from 1000 replicates, are shown at the nodes. Only type strains are included; GenBank accession numbers (in parentheses) are provided for each strain. The *C. magnus* and *C. porcelli* sp. nov. type strains contain two different 16S rRNA gene sequences, and both are included in the tree for each strain. The *Helicobacter pylori* type strain was used to root the tree. The scale bar represents nucleotide sequence divergence.

**Fig. 3.**
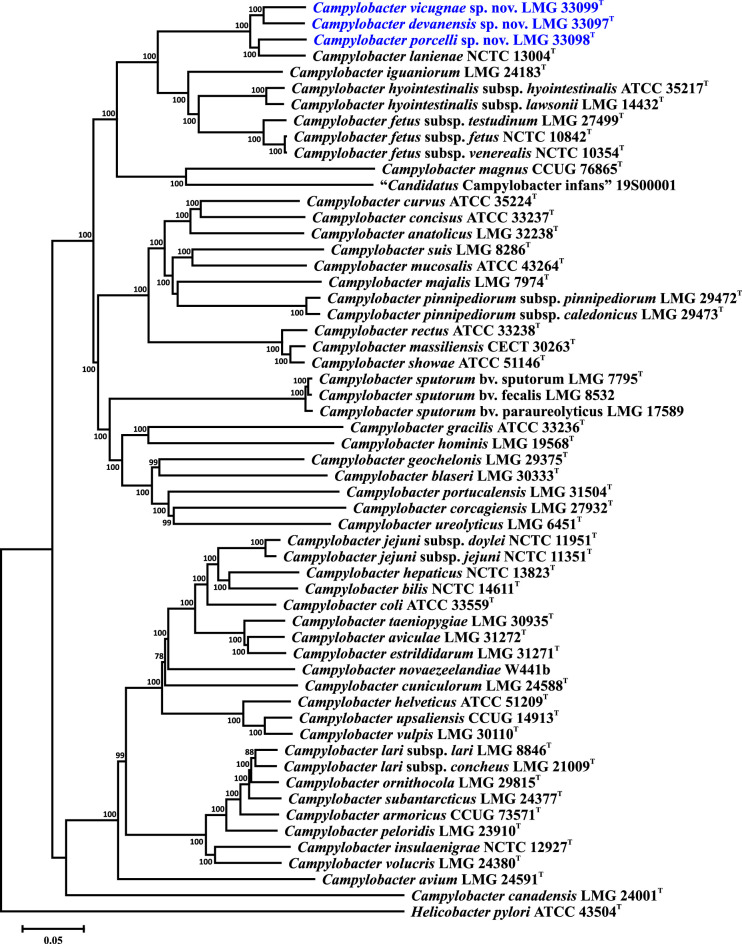
Core genome phylogeny of *Campylobacter devanensis* sp. nov., *Campylobacter porcelli* sp. nov., and *Campylobacter vicugnae* sp. nov. within the genus *Campylobacter*. The phylogeny is based on the concatenated nucleotide sequence alignment of 330 core genes. Only type or reference *Campylobacter* strains are included. The dendrogram was reconstructed using the neighbour-joining algorithm [32] and distances were computed using the Kimura two-parameter model [33]. Bootstrap values of >75%, generated from 1000 replicates, are shown at the nodes. The *Helicobacter pylori* type strain was used to root the tree. The 330 core loci and the corresponding locus tags for all 57 strains are listed in Table S3. The scale bar represents nucleotide sequence divergence.

As described above, the taxonomic position of the three novel selenonegative taxa was first determined based on the single-gene phylogeny of *atpA*. Additional multi-gene analyses were also performed [22] that were limited to the species of the *C. fetus* group and used *C. concisus* as a root. In this study, we performed two additional phylogenetic analyses based on the 16S rRNA gene and the concatenated nucleotide sequences of 330 core genes, respectively (for the core gene list, see Table S3). Each analysis was performed on two sets of strains. The first set included the three proposed type strains and the type or reference strains for all 53 current validly described or Candidatus *Campylobacter* taxa. The second set included all 54 novel strains (where available) and the *C. fetus* group type or reference strains. In all analyses, the *Helicobacter pylori* strain ATCC 43504^T^ was used as a root. 16S rRNA gene alignments were performed using Clustal X and core gene sequence alignments were performed using muscle [30] in Geneious (version 2022.0.1). Phylogenetic trees were reconstructed within mega version 6.06 [31] using the neighbour-joining method [32], the Kimura two-parameter distance estimation method [33] and 1000 bootstrap replicates. Multiple 16S rRNA gene variants of *C. porcelli* and *C. magnus* were included in the analysis but did not noticeably affect the tree topology (Figs 2 and S3). The 16S rRNA gene analysis using the type strains indicated that the novel selenonegative strains could be distinguished from other *Campylobacter* species (Fig. 2). However, the previously described discrete clades observed in the phylogenetic trees based on MLST loci and the core genes were not present here when additional *C. lanienae* and novel selenonegative strains were included (Fig. S3). *C. devanensis* strains were noticeably variable and could not be distinguished from *C. lanienae*. Moreover, the 16S rRNA genes of two *C. devanensis* strains (P0111 and P0136) were substantially different, with the 16S rRNA gene of P0111 clustering with *C. magnus*. However, it has been shown that species identification based on the 16S rRNA gene is unreliable in *Campylobacter* since some species, notably *C. jejuni* and *C. coli*, often cannot be distinguished using this approach [28,34, 35]. Thus, as stated above, we also typed the novel strains using an expanded set of 330 core genes. Similar to the 16S rRNA gene typing of the *Campylobacter* type strains, core genome phylogeny demonstrated that the novel taxa could be readily distinguished from the other *Campylobacter* species (Fig. 3). Of note, the novel selenonegative strains still formed discrete clades when additional strains were included in the analysis, and *C. devanensis* strains did not cluster with either *C. magnus* or *C. lanienae* (Fig. S4).

## Average nucleotide identity and digital DNA–DNA hybridization analyses

Taxonomic placement of *C. devanensis*, *C. porcelli*, and *C. vicugnae* was also performed using average nucleotide identity (ANI) [36] and digital DNA–DNA hybridization (dDDH) [37] analyses. The dDDH value of 70 % for species delineation is approximately equivalent to an ANI value of 95 % [36,38]; however, in *Campylobacter*, some subspecies show pairwise ANI values of 91–95 % (Table 2), while maintaining dDDH values of >70 %. ANI analyses were performed using JSpecies (version 1.2.1) [38]. All pairwise intertaxon ANI values between the three proposed type strains and type strains from validly described and candidate *C. fetus* group taxa were <95 % (Table 2), while all pairwise intrataxon ANI values within the three novel taxa were ≥97 % (Table S4). dDDH analyses were performed using the Genome-to-Genome Distance calculator (GGDC; version 2.1 https://ggdc.dsmz.de/ggdc.php [39,40]). The pairwise dDDH values between the three clades and all other *Campylobacter* taxa were <64 % (Tables 2 and S5) and were thus below the species delineation threshold and consistent with the ANI analysis. GGDC formula 3 was used for all calculations, as recommended by the latest minimal standards for *Campylobacter* [26]. GGDC formula 2 is recommended for analysing draft genomes [37]. Only one of the genomes in Table 2 (i.e., *C. magnus* CCUG 76865^T^) was represented by a draft genome. Nevertheless, no pairwise dDDH values>34 % were found using GGDC formula 2 (data not shown). The ANI and dDDH analyses indicate that the three clades each represent distinct novel species within the genus *Campylobacter*, since the values from both analyses are below the species delineation thresholds.

**Table 2.** Pairwise average nucleotide identity (ANI) and digital DNA–DNA hybridization (dDDH) values (%) between *Campylobacter devanensis* sp. nov., *Campylobacter porcelli* sp. nov., *Campylobacter vicugnae* sp. nov. and related *Campylobacter* strains

ANI	1	2	3	4	5	6	7	8	9	10	11	12
1, *C. devanensis* LMG 33097^T^	–	83.3	86.3	87.0	71.7	71.5	73.9	72.0	71.7	71.7	68.3	68.6
2, *C. porcelli* LMG 33098^T^	83.4	–	83.0	85.9	71.6	71.8	73.1	71.9	71.7	71.7	68.0	68.5
3, *C. vicugnae* LMG 33099^T^	86.5	82.9	–	82.3	71.9	71.7	72.6	72.4	72.0	72.0	68.1	68.5
4, *C. lanienae* NCTC 13004^T^	87.1	86.0	82.4	–	71.5	71.9	74.0	72.0	71.7	71.8	68.5	68.6
5, *C. iguaniorum* LMG 28143^T^	71.8	71.9	72.0	71.6	–	75.4	75.4	75.5	75.0	75.0	68.2	68.2
6, *C. hyointestinalis hyointestinalis* ATCC 35217^T^	71.6	72.0	71.6	71.9	75.4	–	**94.4**	79.3	78.0	78.0	67.8	67.7
7, *C. hyointestinalis* lawsonii LMG 14432^T^	74.1	73.5	72.7	74.1	75.3	**94.3**	–	78.2	77.4	77.9	68.1	68.2
8, *C. fetus testudinum* LMG 27499^T^	72.0	72.0	72.2	71.9	75.4	79.2	78.1	–	**91.9**	**92.0**	67.5	67.7
9, *C. fetus fetus* NCTC 10842^T^	71.9	71.9	72.1	71.9	75.0	78.1	77.5	**91.9**	–	**99.4**	67.6	67.9
10, *C. fetus venerealis* NCTC 10354^T^	72.0	71.9	72.2	71.9	75.0	78.1	78.0	**92.1**	**99.4**	–	67.6	67.9
11, *C. magnus* CCUG 76865^T^	68.1	67.8	67.6	68.2	67.9	67.4	67.7	67.1	67.0	66.9	–	69.4
12, ‘*Candidatus* Campylobacter infans’ 19S00001	68.7	68.7	68.4	68.5	68.1	67.7	68.2	67.9	67.9	67.9	69.9	–
	**LMG 33097^T^**			**LMG 33098^T^**			**LMG 33099^T^**					
**dDDH**	**DDH**	**Model3 C.I.**		**DDH**	**Model3 C.I.**		**DDH**	**Model3 C.I.**				
*C. devanensis* LMG 33097^T^	–	–		52.7	[49.6–55.8]		62.9	[59.6–66.1]				
*C. porcelli* LMG 33098^T^	52.7	[49.6–55.8]		–	–		49.5	[46.4–52.5]				
*C. vicugnae* LMG 33099^T^	62.9	[59.6–66.1]		49.5	[46.4–52.5]		–	–				
*C. lanienae* NCTC 13004^T^	63.6	[60.3–66.8]		61.5	[58.2–64.7]		50.7	[47.6–53.8]				
*C. iguaniorum* LMG 28143^T^	15.4	[12.9–18.3]		16.0	[13.4–18.9]		15.4	[12.9–18.3]				
*C. hyointestinalis hyointestinalis* ATCC 35217^T^	15.3	[12.8–18.2]		15.6	[13.0–18.5]		15.3	[12.8–18.1]				
*C. hyointestinalis lawsonii* LMG 14432^T^	18.5	[15.8–21.5]		17.8	[15.1–20.7]		16.9	[14.3–19.8]				
*C. fetus testudinum* LMG 27499^T^	15.6	[13.1–18.5]		15.6	[13.1–18.5]		15.6	[13.1–18.5]				
*C. fetus fetus* NCTC 10842^T^	15.6	[13.1–18.5]		15.8	[13.2–18.7]		16.0	[13.4–18.9]				
*C. fetus venerealis* NCTC 10354^T^	15.5	[13.0–18.4]		15.7	[13.1–18.6]		16.0	[13.5–18.9]				
*C. magnus* CCUG 76865^T^	13.6	[11.2–16.3]		13.5	[11.2–16.3]		13.3	[11.0–16.1]				
‘*Candidatus* Campylobacter infans’ 19 S00001	13.4	[11.0–16.1]		13.4	[11.0–16.2]		13.3	[10.9–16.1]				

ANI values above 90 % are in bold.

## Geographic range, host range and pathogenicity

Strains from all three putative novel species were isolated in the United States and Scotland, with *C. devanensis* and *C. porcelli* also recovered from pigs in Slovenia. In a study that investigated the epidemiology of campylobacteriosis in Scotland [41,42], 6423 *Campylobacter* isolates were recovered from human clinical samples, or faecal samples from food animals and birds. Strains comprising the three novel species discussed in this study were recovered from swine (37/235; 15.7 %) and from one sheep (1/607; 0.16 %); however, these novel taxa were not recovered from human clinical samples (*n*=3725), cattle (*n*=336), chickens (*n*=1470), or wild birds (*n*=50). Terefe *et al*. [43] reported that strains similar to the type strains of all three taxa were present in the stools of children in rural Eastern Ethiopia, suggesting a large geographic range for these taxa, which would comprise at least North America, Europe and Africa. It is likely that the children in that study acquired these strains by zoonotic transmission from livestock in the area. Livestock in rural Eastern Ethiopia includes sheep, goats, and cattle, consistent with the host range reported here for *C. vicugnae*. However, in this study, *C. devanensis* and *C. porcelli* were recovered exclusively from pigs and wild boars. Since livestock in the study by Terefe *et al*. [43] did not include pigs, it is likely that the host range for these two species includes additional animal species or environmental sources.

Carriage of *C. lanienae* and the three novel taxa described here was >40 % in some kebeles in Eastern Ethiopia [43], and these taxa also often co-occurred with other *Campylobacter* species such as *C. hyointestinalis*, *C. sputorum*, *C. fetus*, *C. ureolyticus*, *C. curvus*, *C. showae*, *C. gracilis*, and *C. pinnipediorum*. Diarrhoea was reported in 48 % of the children by Terefe *et al*. [43]; nevertheless, no positive correlation was found between *Campylobacter* carriage and human disease. Therefore, it is uncertain if the three novel taxa described here are pathogenic. Analysis of the three type strain genomes and some of the draft genomes identified genes often associated with virulence in *Campylobacter*. Strains from all three taxa are motile and contain *cadF* [44], *iam* (*mlaDEF*) [45], *ciaB* [46] and *flpA* [47]. In addition, *C. devanensis* and *C. porcelli* encode SodC, which protects against superoxides produced by macrophages and is important for virulence [48,49]. Some *C. vicugnae* strains also contain the *zot* gene encoding zonula occludens toxin [50]. Cytolethal distending toxin-encoding loci (*cdtABC*) were identified in all three taxa. Notably, multiple *cdt* loci in the same genome were identified in *C. porcelli* and *C. vicugnae*; however, in *C. vicugnae*, the *cdt* loci were degenerate in some strains. Although the gene content of these strains indicates that some strains may be virulent, more research is necessary to determine if these proposed species, or strains within these species, can cause human illness.

## Conclusion

The results of the present polyphasic study clearly indicate that the *C. lanienae*-associated selenonegative strains belong to three novel *Campylobacter* species. Core genome phylogenetic analysis shows that these strains form three distinct clades within the genus *Campylobacter*, ANI and dDDH values are all are below the species delineation thresholds, and phenotypic analyses revealed unique phenotypic profiles within the genus. Although strains of these novel species have also been reported in faecal samples from human patients with diarrhoea, the pathogenicity of these novel species is uncertain and should be further investigated. Strains recovered from pigs formed two separate species, for which the names *Campylobacter devanensis* sp. nov. and *Campylobacter porcelli* sp. nov. are proposed. Strains recovered from small grazing animals (e.g., sheep, goats, and alpacas) form a third species, for which the name *Campylobacter vicugnae* sp. nov. is proposed.

## Description of *Campylobacter devanensis* sp. nov.

*Campylobacter devanensis* (de.van.en’sis. L. masc. adj. *devanensis*, of *Devana*, the presumed Roman name for Aberdeen, where the strains were isolated).

Cells are Gram-negative, spiral, and motile. Coccoid cells are present in some cultures. After 72 h culture at 37 °C under microaerobic conditions on ABA-B, colonies are glistening, opaque, convex and circular with entire margins and are 1–2 mm in diameter. Growth occurs on ABA-B at both 37 and 42 °C under microaerobic conditions and at 37 °C under anaerobic conditions. No growth on ABA-B at 30 °C under microaerobic conditions or at any temperature under aerobic conditions. All strains demonstrate oxidase and catalase activity but no urease, hippuricase or α-haemolytic activity. All strains demonstrate alkaline phosphatase activity and hydrolyse indoxyl acetate. All strains reduce nitrate, selenite and TTC. Growth is supported on ABA-B with a final NaCl concentration of 2 % (w/v) and on ABA-B supplemented with 1 % (w/v) glycine, but not on ABA-B supplemented with 0.04 % (w/v) TTC. Strains are resistant to 30 mg l^−1^ cephalothin and to 30 mg l^−1^ nalidixic acid. All strains were recovered from swine. The type strain, RM3662^T^ (=LMG 33097^T^=NCTC 15074^T^), was isolated from pig faeces in 1994. Accession numbers for the 16S rRNA gene and genome sequence of the type strain are OQ561783 and CP018788, respectively.

## Description of *Campylobacter porcelli* sp. nov.

*Campylobacter porcelli* (por.cel'li. L. gen. n. *porcelli*, of a little pig).

Cells are Gram-negative, motile, curved rods. Coccoid cells are present in some cultures. After 72 h culture at 37 °C under microaerobic conditions on ABA-B, colonies are glistening, opaque, convex and circular with entire margins and are 1–2 mm in diameter. Growth occurs on ABA-B at both 37 and 42 °C under microaerobic conditions and at 37 °C under anaerobic conditions. No growth on ABA-B at 30 °C under microaerobic conditions or at any temperature under aerobic conditions. All strains demonstrate oxidase and catalase activity but no urease, hippuricase or α-haemolytic activity. All strains demonstrate alkaline phosphatase activity, but do not hydrolyse indoxyl acetate. All strains reduce nitrate, selenite and TTC. Growth is supported on ABA-B with 2 % (w/v) NaCl and on ABA-B supplemented with 1 % (w/v) glycine, but not on ABA-B supplemented with 0.04 % (w/v) TTC. Strains are resistant to 30 mg l^−1^ cephalothin and to 30 mg l^−1^ nalidixic acid. All strains were recovered from swine. The type strain, RM6137^T^ (=LMG 33098^T^=CCUG 77054^T^=NCTC 15075^T^), was isolated from wild boar faeces in 2006. Accession numbers for the 16S rRNA gene sequences of the type strain are OQ561780 and OQ561781. The accession number of the type strain genome sequence is CP018789.

## Description of *Campylobacter vicugnae* sp. nov.

*Campylobacter vicugnae* (vi.cug’nae. N.L. gen. n. *vicugnae*, of *Vicugna pacos*, the source of the type strain).

Gram-negative cells are motile with curved to spiral morphology. Coccoid cells are present in some cultures. After 72 h culture at 37 °C under microaerobic conditions on ABA-B, colonies are glistening, opaque, convex and circular with entire margins and are 1–2 mm in diameter. Growth occurs on ABA-B at both 37 and 42 °C under microaerobic conditions and at 37 °C under anaerobic conditions. No growth on ABA-B at 30 °C under conditions or at any temperature under aerobic conditions. All strains have oxidase and catalase activity but no urease, hippuricase or α-haemolytic activity. All strains have alkaline phosphatase activity, but do not hydrolyse indoxyl acetate. All strains reduce nitrate and TTC, but not selenite. Growth is not supported on ABA-B with a final NaCl concentration ≥2 % (w/v), ABA-B supplemented with 1 % (w/v) glycine, or on ABA-B amended with 0.04 % (w/v) TTC. Strains are resistant to 30 mg l^−1^ nalidixic acid; resistance to 30 mg l^−1^ cephalothin is variable. Strains were recovered from alpacas, goats, and sheep. The type strain, RM12175^T^ (=LMG 33099^T^=CCUG 77055^T^=NCTC 15076^T^), was isolated from alpaca faeces in 2010. Accession numbers for the 16S rRNA gene and genome sequence of the type strain are OQ561782 and CP018793, respectively.

## Abbreviations

ABA: anaerobe basal agar
ABA-B: anaerobe basal agar with blood
ANI: average nucleotide identity
dDDH: digital DNA-DNA hybridization
IA: indoxyl acetate
IVS: intervening sequence
mCCDA: modified charcoal-cefoperazone-deoxycholate agar
MLST: multilocus sequence typing
TSI: triple sugar iron
TTC: triphenyltetrazolium chloride
